# Dynamic Change of CD34 Level during the Survival Process of Narrow Pedicle Flap

**DOI:** 10.1371/journal.pone.0142417

**Published:** 2015-11-11

**Authors:** Lijun Wu, Tianlan Zhao, Daojiang Yu, Qi Chen, Wenya Han, Wenyuan Yu, Wei Sun

**Affiliations:** Department of Plastic and Cosmetic Surgery, the Second Affiliated Hospital of Soochow University, Suzhou 215004, China; Centro Cardiologico Monzino, ITALY

## Abstract

**Objective:**

To evaluate the dynamic change of CD34 level during the survival process of narrow pedicle flaps.

**Methods:**

Twenty-five white pigs were randomly and equally divided into 5 experimental groups. Five different type of narrow pedicle with different length-to-width ratio were employed, and each type of narrow pedicle was covered with 5 different size random flaps and which was classified into A, B, C, D and E for 5 groups. Group A was control group. Each type narrow pedicle with 5 different skin flaps were implanted onto the back of the pigs along the midline of back with a reverse direction. A 0.3 cm×0.3 cm full thickness skin flap in the middle of distal segment was collected and on 3rd, 5th, 7th and 14th days of post-operation. The expression of CD34 was measured by immunohistochemistry and enzyme-linked immunosorbent (ELISA).

**Results:**

Histological examination showed that with the increasing of length-to-width ratio of the narrow pedicle skin flaps, the expression of CD34 increased in the skin flaps. Increased level of CD34 was found on 3rd day post-operation, and the peak expression was found on 7th day. Persistent high level of CD34 was found until 14th day.

**Conclusion:**

Increased CD34 level in the distal skin flap, there is the association between CD34 level and ischemia injury. Moreover, CD34 expression plays an important role during the repair processes of pedicle flaps.

## Introduction

Random flap is the most commonly used flap in tissue repair. The clinical application of these flaps were influenced by limitation of length-to-width ratio for a long time[1]. Recently, several studies have evaluated the random flaps in multiple models and the results were satisfaction [2–4].

With the further study of the narrow pedicle flap [5–7], safe and efficacy outcome could be achieved by using skin flaps. However, the detail mechanism indicates that it could influence the survival of the pedicle skin flap. To our knowledge, the mechanism has not been explored yet. CD34 is a key factor during the angiogenesis and might be involved in the process of flap survival.

Here, we tried to explore the relationship between the length-to-width ratio of pedicle and skin survival area of random flaps, and evaluated the dynamic change of CD34 during the survival process of narrow pedicle flap.

## Materials and Methods

### Experimental animals and ethics statement

25 white pigs were purchased from the Experimental Animal Center of Soochow University, weight 25–30 kg. The study protocol was also approved by the Institutional Animal Care and Use Cotines for animal experimentation. All surgery was performed under sodium pentobarbital anesthesia, and all efforts were made to minimize suffering. The pigs were slaughtered two weeks after the operation with anesthesia.

### Animal grouping and flap allocation

25 pigs were randomly divided into 5 groups (named as I-V group). The length-to-width ratio of pedicle was 0:2 cm (Group I); 1 cm: 2 cm (Group II); 2 cm: 2 cm (Group III); 3 cm: 2 cm (Group IV) and 4 cm: 2 cm (Group V), and each narrow pedicle was covered with 5 different random skin flap and areas were as follows: 2 cm × 2 cm (A); 3 cm × 3 cm (B); 4 cm × 4 cm (C); 5 cm × 5 cm (D) and 6 cm × 6 cm (E). The A, B, C, D and E skin flaps were implanted onto the back of each pig. The interval space of flaps, between pedicle and midline of the back was 4 cm. All the flaps were arranged with reverse direction on the back of pigs.

### Pedicle skin flap implantation

The flap was removed from the superficial layer of the deep temporal fascia and sutured with basement tissue after achievement of hemostasis. The flap was packed with a topical dressing and a compression bandage was applied underneath the abdominal belt. After surgery, pigs were breed separately and administrated with antibiotic (Intramuscular injection of 800,000 unit/times penicillin, twice daily) for 1 week.

### Specimen collection

A 0.3 cm× 0.3 cm full thickness skin flap in the middle of distal segment of each pig was collected at intraoperative time, and on 3rd, 5th, 7th and 14th days of post-operation. Each specimen was divided into 2 parts. One was fixed in 10% formaldehyde for routine hematoxylin-eosin (H&E) staining and immunohistochemistry analysis of CD34 expression. Another one was stored in-80try analysis on 3rd, 5th, 7th and 14th days of post-operation.

### Observation indexes

Observation of physical characteristics was performed at every 24 h postoperation and the pigs were color, swelling, capillary filling, infection and necrosis of flaps were evaluated and recorded by photos. The survival of skin flap was assessed on 14th day and surface area of survival tissue was measured by using standard grid paper.

Qualitative analysis of CD34 expression were carried out using commericial available histological kit from Life Techonology (Carlsbad, CA) according to the manufacturer's instruction, where the quantitative analysis of CD34 was performed by using enzyme linked immunosorbent (ELISA) kit purchased from R&D company(Miniapolis, MN).

### Statistical analysis

All the experiments were performed triplicated to obtain consistent results and the statistical analysis was carried out by using SPSS 17.0 (SPSS Inc. Chicago, IL). The data was expressed as mean± standard error (SE). Multiple group comparison was performed by using one way ANOVA analysis, followed by Newnan-Keuls test for further analysis of between group results. P<0.05 was considered as statistical significance.

## Results

### General observation indexes and flap survival

**Fig 1:**
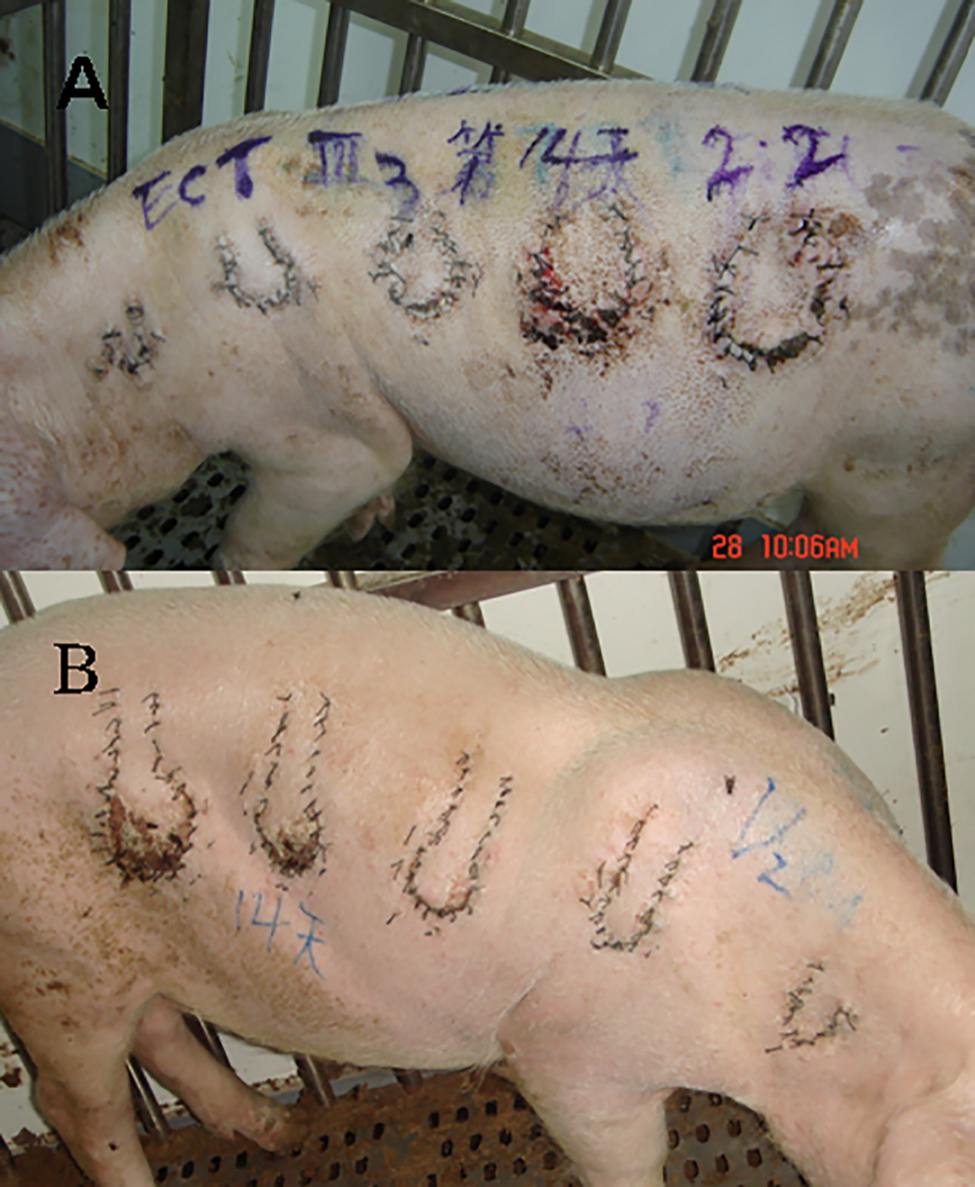
The survival of flaps. A) After 14 days all flaps of one pig in group III completely survived. B) After 14 days in group V the distal part of flaps D and E were necrotic. Plantar local **random flaps** provide a versatile method of plastic coverage for many types of pedal soft tissue defects. The ability of a local **random flap** to replace "like with like tissue" on the weight-bearing surface of the forefoot makes it a useful option for coverage of recalcitrant submetatarsal ulcerations. The authors describe a technique of adjunctive temporary stabilization of the affected metatarsophalangeal joint to maximize the likelihood of ultimate flap survival.

On the 14th day postoperation, flaps in Group I, II and III were all survived while Flap A, B, C in Group IV and V were all survived. With the increasing of the flap area, necrosis was found in Flap D and E in IV and V groups. However, no reduction was found on the survival area of the flaps in IV and V groups (Fig 1). In the flaps with same sizes, we did not observe a difference on the survival area in the pedicles with different length/width ratio. The survival area of flap A was 3.14 cm^2^, flap B was 7.07 cm^2^, and flap C was 12.56 cm^2^. However, when the size of the flap reached 5×5, the survival area of the same flap was reduced. The survival area of flap D was 19.63cm^2^ in Group I, II, III, and flap D was (18.56±0.76)cm^2^ in group IV, and flap D was (18.35±0.65)cm^2^ in group V. The survival area of flap E was 28.26cm^2^ in I, II, III groups and flap E was (18.55±0.56)cm^2^ in group IV, and flap E was (18.34±0.45) cm^2^ in group V. The detail results were shown in Table 1. Significant differences were found on the survival area of flaps between groups (p<0.05).

**Table 1:** The postoperation survival area of the flaps in each group (Mean±SD, cm^2^).

Group	n	A (2×2)	B (3×3)	C (4×4)	D (5×5)	E (6×6)
I 2:0	10	3.14±0.00	7.07±0.00	12.56±0.00	19.63±0.00	28.26±0.00
II 2:1	10	3.14±0.00	7.07±0.00	12.56±0.00	19.63±0.00	28.26±0.00
III 2:2	10	3.14±0.00	7.07±0.00	12.56±0.00	19.63±0.00	28.26±0.00
IV 2:3	10	3.14±0.00	7.07±0.00	12.56±0.00	18.56±0.76	18.55±0.56
V 2:4	10	3.14±0.00	7.07±0.00	12.56±0.00	18.35±0.65	18.34±0.45

Flap A was set as the control, and p<0.05 when compare with flap A group.

### Immunohistochemical analysis of CD34 expression

**Fig 2:**
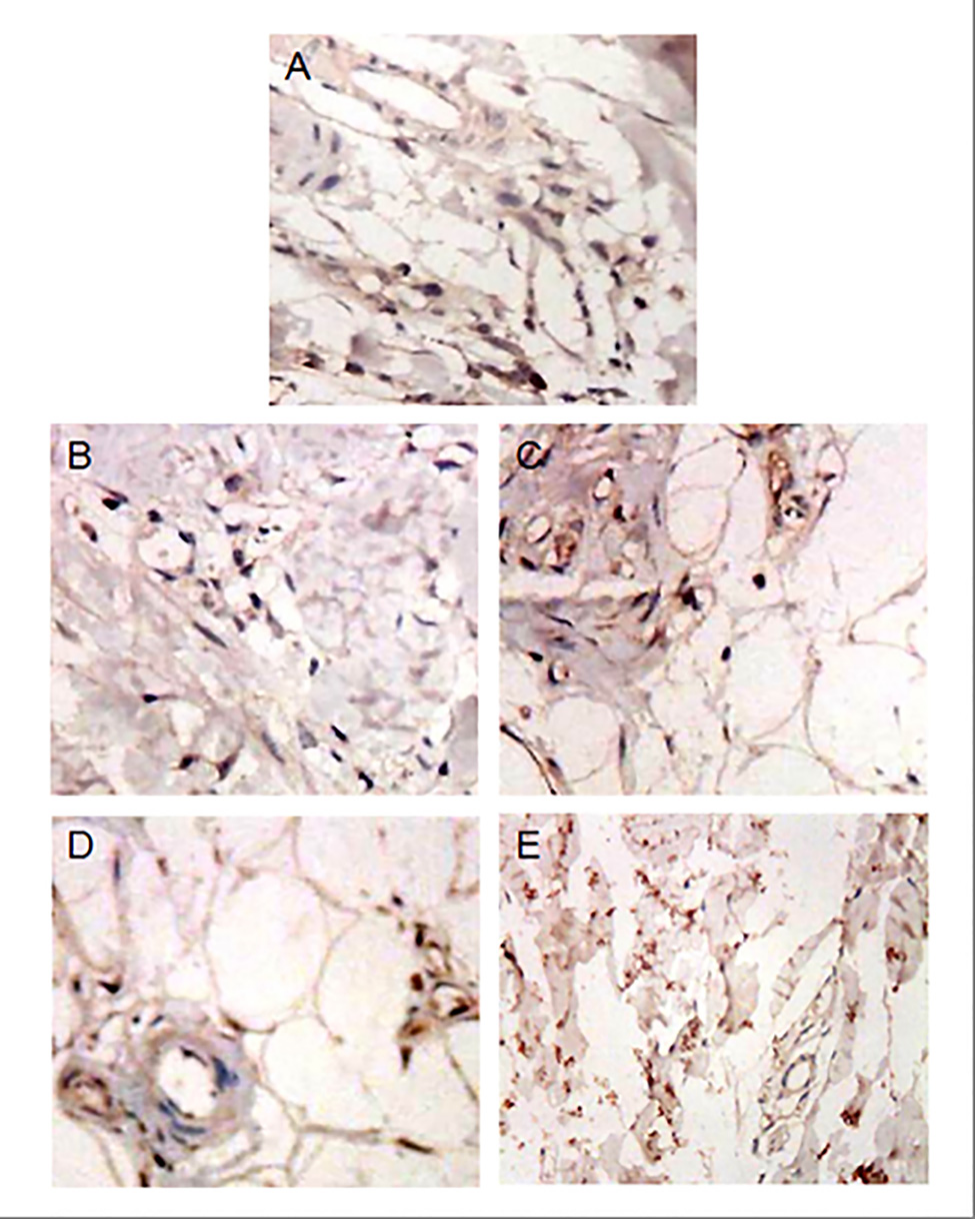
The expression of CD34 in flap tissues at different time points. (A). Intraoperation, (B). 3rd day postoperation, (C). 5th day postoperation, (D). 7th day postoperation, (E). 14th days postoperation. ×400 magnification. All the images were from the flap E in Group V.

CD34 antigen is mainly expressed in the lumen of capillary, protrusion of the cell membrane and the cross area between the endothelial cells. The CD34 positive staining on the flap tissue showed brown particle. According to the immunohistochemical results, only a few brown particles were found in the flap obtained at the intraoperative time points. After operation, increased expression of CD34 was found with the prolonged time, and the peak staining of CD34 was observed on 7th day postoperation and maintained in high level until 14th day postoperation (Fig 2).

### Quantitative analysis of CD34 expression by ELISA

We here only showed the results from groups III and V which are the most representative data to explain the data, and the results were shown in Tables 2, 3 and 4. The ELISA results were consistent with the immunohistochemical analysis. Increased CD34 level was found 3rd day after operation, and reached peak at 7th day after operation. Sustained high level CD34 expression was found at 14th day after operation (**P <0.05**) (Table 2 and Table 3). Significantly increased level of CD34 was found from 3rd day to 14th day in group V than in group III.

**Table 2:** The expression of CD34 in different flap at different time points in group III (Mean±SD, IU / ml).

flap	n	Day 0	Day 3	Day 5	Day 7	Day 14
A	10	6.507±0.392	7.201±1.04	7.348±1.07	9.507±1.36	9.447±1.23
B	10	6.406±0.356	7.266±1.12	7.409±1.13	9.611±1.31	9.467±1.38
C	10	6.527±0.437	7.374±1.09	7.453±1.09	9.680±1.29	9.540±1.25
D	10	6.706±0.342	7.380±1.08	7.513±1.26	9.790±1.09	9.682±1.02
E	10	6.631±0.328	7.470±1.16	7.522±1.21	9.798±1.17	9.702±1.27

Note: Increased CD34 level was found 3rd day after operation, and reached peak at 7th day after operation. Sustained high level CD34 expression was found at 14th day after operation (**P <0.05**).

**Table 3:** The expression of CD34 in different flap at different time points in group V(Mean±SD, IU / ml).

flap	n	Day 0	Day 3	Day 5	Day 7	Day 14
A	10	6.502±0.382	8.813±0.463	10.430±0.613	10.760±0.615	10.014±0.793
B	10	6.405±0.366	9.581±0.507	10.631±0.711	11.301±0.718	11.201±0.613
C	10	6.517±0.537	9.628±0.662	10.784±0.705	11.484±0.548	11.413±0.761
D	10	6.606±0.332	9.781±0.573	11.300±0.618	11.536±0.602	11.522±0.811
E	10	6.621±0.318	9.709±0.605	11.257±0.725	11.525±0.697	11.480±0.502

Note: Increased CD34 level was found 3rd day after operation, and reached peak at 7th day after operation. Sustained high level CD34 expression was found at 14th day after operation (**P <0.05**).

**Table 4:** Comparison the CD34 level in different flat in Group III and group V at different time points (Mean±SD, pg/ml).

flap	Day 0		Day 3		Day 5		Day 7		Day 14	
	III	V	III	V	III	V	III	V	III	V
A	6.507	6.502	7.201	8.813*	7.348	10.430*	9.507	10.760*	9.447	10.014*
B	6.406	6.405	7.266	9.581*	7.409	10.631*	9.611	11.301*	9.467	11.201*
C	6.527	6.517	7.374	9.628*	7.453	10.784*	9.680	11.484*	9.540	11.413*
D	6.706	6.606	7.380	9.781*	7.513	11.300*	9.790	11.536*	9.682	11.522*
E	6.631	6.621	7.470	9.709*	7.522	11.257*	9.798	11.525*	9.702	11.480*

* p<0.05 when compared with group III. The expression of CD34 on 3rd day and 5th day postoperation in groups III and V was significantly higher. The level of CD34 reached peak on 7th day postoperation in groups III and V, and reached baseline level on 14th day postoperation.

## Discussion

### Survival processes of the flaps

Necrosis of ischemic tissue remains the main and troublesome complication in skip flap surgery. No effective therapy is currently available due to the in determined pathogenesis. During the past few years, many researchers have made efforts to elucidate the underlying mechanisms, vasospasm and arteriovenous shunt flow [8–13]. There are 3 stages were involved in the processes of flap revascularization, including capillary bud formation, granulation and microcirculation tissue formation, and modified vascular tissue formation. No literature sure that the length/width ratio of pedicles was not a key factor for flap survival.

Basis on the results, setting of same length-to-width of pedicle and increasing survival area was found with the flap area increasing. However, when the length/width ratio of pedicle was at least 2:3, necrosis tissues could be found in the 5cm×5cm and 6cm×6cm flaps. But we have not observed a reduction on survival area of the flap. According to a series of research findings and clinical applications, there is no relationship between the duration of flap survival and the width of the pedicle. The length/width ratio should no be considered as the most important standard measure. More accurate methods for the design of flap pedicles include the method that Taylor. And others have adopted, or other methods such as Doppler.

### The significance of CD34 level

CD34 is usually employed as surface marker of primitive hematopoietic stem cell, and the expression of CD34 is also found in the vascular endothelial cells, which play an important role in the angiogenesis. Highly expression of CD34 was found on the proliferated vascular endothelial cells. Regardless of the exact mechanisms of CD34 in the process of angiogenesis, the expression of CD34 on the endothelial progenitor cells (EPCs) and endothelial cells could reflect the density of blood vessels in the tissue [14–21].

In this study, we found that the expression of CD34 on 3rd day and 5th day postoperation in groups III and V was higher than other flaps. The level of CD34 was decreased on 7th day postoperation in groups III and V, and reached baseline level on 14th day postoperation. With the increasing of the length-to-width ratio of pedicles, the CD34 level was increased in each flap tissue, and we observed a greatly increasing level of CD34 in group V than that in group III. Moreover, in the same size of pedicle, the expression of CD34 was increased with the skin flap up. The injury tissue could induce to increase the expression of CD34, which play an important role in the wound healing, and with the healing of the flap and skin, the expression of CD34 gradually decreased. However, keeping high level of CD34 was found in the Flap D and E of group V, which could be caused by severe inflammatory which was derived from necrosis tissue of distal flap.

Therefore, we suggested that CD34 plays a critical role in the different length-to-width ratio of pedicle, which might influence the flap repair. We observed that with the increasing of the flap area, the flap CD34 content gradually increased, which indicate that increasing number of blood vessels in the tissue, and our results challenge the traditional view that the length-to-width ratio of pedicle could affect the flap survival. The expression of CD34 was not significantly change until pedicle reached or larger than 2:3 and the flap size was 5cm×5cm or larger.

### The advantages of narrow pedicle flaps

Pedicle flap has been a hot area in the plastic surgery. A pedicle with suitable length-to-width ratio could facilitate the flap monitoring, which is similar to the island skin flap. We also challenged length/width ratio of the pedicle vs. traditional random flaps at some degree. The application of this kind of pedicle flap in clinical practice also showed satisfactory results.

In this experimental study, we found that CD34 level increasing was found 3rd day after operation, and reached peak at 7th day after operation. Sustained high level CD34 expression was found at 14th day after operation, which was consistent with the studies. CD34 was involved in angiogenesis process during the wound healing [22–24]. The use of exogenous CD34 intervention might be considered in the clinical practice. However, further studies are still needed to optimize the length-to-width ratio of pedicle before the application for the human subjects.

## Supporting Information

S1 File The raw data of CD34 level in different flat in group III and group V at different time points. (XLS)
